# Mechanism of Anti‐Cancer in Breast Cancer Cells With HER2 Overexpression by Dietary Supplement of Five Edible Plants

**DOI:** 10.1002/fsn3.70617

**Published:** 2025-07-14

**Authors:** Pornnapa Sitthisuk, Nichakarn Kwankaew, Sukanda Innajak, Kanokwan Nilchad, Watcharaporn Poorahong, Patiwat Kongdang, Ramida Watanapokasin

**Affiliations:** ^1^ Department of Biochemistry Faculty of Medicine, Srinakharinwirot University Bangkok Thailand; ^2^ Department of Basic Medical Science Faculty of Medicine Vajira Hospital, Navamindradhiraj University Bangkok Thailand; ^3^ Department of Biochemistry Faculty of Medicine, BangkokThonburi University Bangkok Thailand

**Keywords:** anti‐cancer, apoptosis, breast cancer cell, five edible plants, Mylife/Mylife100 supplement

## Abstract

Edible plants are known to have many pharmacological properties, including anti‐cancer. Mylife/Mylife100, a Thai dietary supplement composed of five types of edible plants ( *Sesamum indicum* L., *Psidium guajava* L., *Centella asiatica* (L.) Urb., *Glycine max* (L.) Merr., and *Garcinia mangostana* L.) was evaluated for its anti‐cancer effect on breast cancer cells with HER2 overexpression (SK‐BR‐3 cell line). The results demonstrated that the supplement showed cytotoxic activity against SK‐BR‐3 cell growth in a dose‐dependent manner, with an IC_50_ value of 116.5 ± 2.30 μg/mL. The effect of the supplement on apoptosis induction in SK‐BR‐3 cells was investigated. The findings revealed that the supplement induced nuclear condensation and loss of mitochondrial membrane potential, both hallmarks of apoptosis. Additionally, the supplement increased the sub‐G1 population analyzed by flow cytometry and was further validated by attenuation of anti‐apoptotic proteins (Bcl‐xL and Mcl‐1) and the induction of pro‐apoptotic proteins (Bax, cleavage caspase 7 and cleavage PARP). Moreover, the supplement also inhibited the PI3K/Akt pathway and enhanced MAPK signaling pathways. These findings indicated that the Mylife/Mylife100 supplement suppresses cell growth and induces apoptosis via activation of the intrinsic pathway and regulation of the PI3K/Akt and MAPK signaling pathways against SK‐BR‐3 cells.

## Graphical abstract

Mylife/Mylife100, a Thai a dietary supplement, composed of five types of edible plants (*Sesamum indicum* L., *Psidium guajava* L., *Centella asiatica* (L.) Urb., *Glycine max* (L.) Merr., and *Garcinia mangostana* L.) was evaluated for their anti‐cancer effect on drug resistant breast cancer cells with HER2 overexpression (SK‐BR‐3 cell line). The supplement showed cytotoxic activity against SK‐BR‐3 cells growth in a dose‐dependent manner. These findings indicated that Mylife/Mylife100 supplement suppresses cell growth and induces apoptosis via activation of the intrinsic pathway and regulation of the PI3K/Akt and MAPK signaling pathways against SK‐BR‐3 cells.

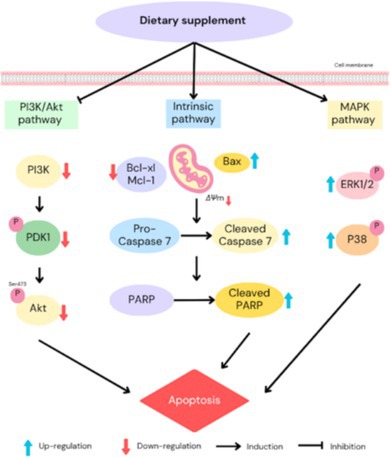

## 1 Introduction

Cancer is a condition characterized by the unchecked growth of abnormal cells in the body. These cells are capable of generating their own growth signals and can avoid the process of programmed cell death, which are major hallmarks of cancer cells (*Fouad and Aanei* 2017). According to data from the National Cancer Institute of Thailand in 2019, breast cancer is the most common cancer in the female, accounting for up to 40% of new cases (“National Cancer Institute. Hospital‐based cancer registry annual report” 2019).

Human epidermal growth factor receptor‐2 (HER2)‐positive breast cancer represents approximately 13% to 15% of all breast cancer case (*Li* et al. 2025). This subtype is characterized by overexpression of HER2 protein, which is associated with a poor prognosis due to its role in promoting cancer cell growth and proliferation (*Cheng* 2024; *Galogre* et al. 2023). Although HER2‐targeted therapies such as trastuzumab and pertuzumab have significantly improved clinical outcomes in patients with HER2‐positive breast cancer, the development of resistance remains a major clinical challenge. Two major intracellular signaling pathways downstream of HER2, namely the PI3K/Akt and MAPK (mitogen‐activated protein kinase) pathways, play crucial roles in mediating tumor cell proliferation, survival, and resistance to apoptosis. Activation of these pathways has been implicated in the development of therapeutic resistance, particularly when alternative signaling cascades are upregulated or when mutations occur in key signaling components. The mechanisms underlying this resistance are complex and multifactorial, involving HER2 gene mutations, activation of alternative signaling pathways such as PI3K/Akt/mTOR, overexpression of other growth factor receptors, and immune evasion (*Geng* et al. 2024; *Li* et al. 2025; *Xu* et al. 2024). These limitations underscore the urgent need for novel therapeutic strategies that can overcome or bypass trastuzumab resistance. Consequently, there is an ongoing need for the identification of novel therapeutic agents to effectively treat or prevent HER2‐positive breast cancer, with particular interest in those derived from medicinal plants as a promising alternative approach. This is because plants and herbs demonstrate low cytotoxicity to normal cells. Furthermore, apoptosis has become an alternative target of cancer research (*Hosseini and Ghorbani* 2015; *Sharma* et al. 2024). This approach is consistent with current chemical drugs, which aim to induce apoptosis in cancer cells (*Pistritto* et al. 2016).

Edible plants are a source of a wide variety of nutrients and bioactive compounds, which provide health benefits and serve as an important source for the development of new drugs to prevent diseases including cancer (*Hossain* et al. 2025; *Zhao* et al. 2015). Mylife/Mylife100, a Thai innovation, is a dietary supplement composed of mangosteen aril ( *Garcinia mangostana* L.), pennywort leaves ( *Centella asiatica* (L.) Urb.), guava fruit ( *Psidium guajava* L.), black sesame seeds ( *Sesamum indicum* L.), and soy protein ( *Glycine max* (L.) Merr.). For more than 10 years, it has served as an alternative treatment for cancer patients in Thailand (Wiriyachitra, et al. 2024). A multitude of studies on these five types of edible plants have reported exhibiting several compounds, such as phenolic compounds, which are widely found in plants and can inhibit or attenuate the initiation, progression, and spread of cancer. These five types of edible plants have demonstrated anti‐cancer activities, including the inhibition of cell growth and the stimulation of apoptosis in many types of cancer cells through various cell signaling pathways (*Ahmad* et al. 2014; *Lok* et al. 2023; *Pedraza‐Chaverri* et al. 2008; *Wiciński* et al. 2024; *Wu* et al. 2019). For example, sesamin, a major lignin from sesame seeds, reduced cell viability in several human breast cancer cells such as the MCF‐7 cell line (*Sohel* et al. 2022), and α‐mangostin from ethanolic extracts of mangosteen induced apoptosis and suppressed migration and invasion through the PI3K/Akt signaling pathway in the breast cancer cell line MDA‐MB‐231 (*Zhu* et al. 2021). Interestingly, this dietary supplement has also shown effectiveness in enhancing immunity, as indicated by significant increases in CD4 and CD8 (killer T cell) levels observed in volunteers. It also promotes telomere lengthening in white blood cells (leukocytes), which may play a role in cancer prevention (Wiriyachitra, et al. 2024). However, studies on the dietary supplement composed of five types of edible plants on breast cancer cells with HER2 overexpression remain to be studied.

Therefore, in this study, we investigated the anticancer effects of a combination of five types of edible plants on cell growth inhibition and apoptosis induction in SK‐BR‐3 cells (HER2 overexpression). The results highlight the potential of the Mylife/Mylife100 supplement against breast cancer cells, suggesting that it may serve as an alternative dietary supplement for the prevention or treatment of breast cancer, either alone or in combination with chemotherapy, offering potential benefits for future therapies.

## 2 Material and Methods

### 2.1 Cell Culture

The breast cancer cell line, SK‐BR‐3, was obtained from the American Type Culture Collection (ATCC, Manassas, VA). Cells were grown in Roswell Park Memorial Institute (RPMI) 1640 medium supplemented with 100 U/mL penicillin, 100 μg/mL streptomycin, and 10% fetal bovine serum (FBS). They were maintained in a CO_2_ incubator at 37°C under 5% carbon dioxide (CO_2_) and saturated humidity (95%). The medium was renewed every 2–3 days.

### 2.2 Preparation of Five Edible Plants

The supplement (dietary supplement product Mylife/Mylife100) was obtained from Asian Phytoceuticals Public Company Limited as mixed powder. The sample was prepared according to a previous report (*Praengam* et al. 2024). Briefly, aril mangosteen, pennywort leaves, guava fruits, black sesame seeds, and soybeans were mixed with water. The mixture was then filtered through a decanter by centrifuging at 1400 rpm. Subsequently, the juice was heated at 70°C for 30 min using a steam boiler, followed by spray drying to obtain a fruit juice powder. Each supplement capsule contains the following active ingredients: 150 mg of pennywort leaf powder (equivalent to 750 mg fresh weight), 100 mg of black sesame seed powder (from 2 g fresh weight), 100 mg of isolated soy protein powder (from 400 mg fresh weight), 100 mg of guava fruit juice powder (from 2 g fresh weight), and 50 mg of mangosteen aril juice powder (from 250 mg fresh weight). The final product was officially registered with the Thai FDA as a dietary supplement. For in vitro experiments, the supplement powder was dissolved in DMSO to prepare a stock solution and then diluted with culture medium to the desired concentrations. The final concentration of DMSO in all treatment and control groups was adjusted to 0.15% (v/v).

### 2.3 Cells Cytotoxicity Assay

The cytotoxicity of the supplement was assessed using the MTT assay. Briefly, SK‐BR‐3 cells were seeded and incubated overnight. Then, the cells were treated with various concentrations of supplement at 0, 7.8125, 15.625, 31.25, 62.5, 125, 250, and 500 μg/mL, whereas the control group was treated with DMSO for 24 h. Following treatment, MTT solution (0.5 mg/mL) was added after the medium was removed. After that, the cells were incubated for 2 h at 37°C. After that, MTT solution was discarded, and DMSO was used to dissolve the MTT formazan crystals. A microplate reader (Multiskan Sky Microplate Spectrophotometer, USA) was utilized to measure the absorbance at 570 nm.

### 2.4 Detection of Nuclear Morphology Changes

To identify the nuclear morphological alterations of apoptotic cells, Hoechst 33342 staining was employed. Hoechst 33342 staining was applied to SK‐BR‐3 treated cells, which were then incubated at 37°C for 30 min in the dark. The images were captured using a fluorescence microscope (Olympus, Japan). ImageJ software (version 1.53e) is utilized to quantify the number of cells in images and to determine the percentage of condensed cells.

### 2.5 Detection of Mitochondrial Membrane Potential ( *ΔΨ*m)

The impact of the supplement on the decrease in mitochondrial membrane potential was examined using JC‐1 staining. SK‐BR‐3 cells were seeded and treated with various concentrations of supplement at 0, 50, 75, 100, 125, and 150 μg/mL for 6 h. The cells were then stained with JC‐1, incubated in the dark for 10 min, washed with PBS, and subsequently observed under a fluorescence microscope (Olympus, Japan). The fluorescence intensity of JC‐1 staining was measured with ImageJ software (version 1.53e).

### 2.6 Cell Cycle Analysis

Flow cytometry was utilized to examine apoptosis induction through the increase of the sub‐G1 population. SK‐BR‐3 cells were seeded and treated with various concentrations of supplement at 0, 50, 75, 100, 125, and 150 μg/mL for 24 h. Then, cells were harvested, washed with PBS, and fixed in ice‐cold 70% ethanol, respectively. After that, cells were stained with Guava cell cycle reagent (Merck Millipore Corp., Merck KGaA) and DNA content was determined by using Guava EasyCyteTM flow cytometer and GuavaSoftTM software (Merck Millipore Corp., Merck KGaA).

### 2.7 Western Blot Analysis

The expression of the protein was detected via Western blot analysis. The protein of cells was extracted using RIPA lysis buffer (50 mM Tris–HCl, pH 7.5, 5 mM EDTA, 250 mM NaCl, 0.5% Triton X‐100), which contained 10 mM PMSF and complete mini protease inhibitor cocktail (Roche Diagnostics GmbH, Mannheim, Germany). SDS‐polyacrylamide gel electrophoresis (PAGE) was used to separate the protein mixture, which was then transferred onto polyvinylidene difluoride (PVDF) membranes. After that, the membranes were blocked for 1 h at room temperature using 5% BSA. The membranes were incubated at 4°C with a primary antibody specific to the target proteins (Cell Signaling Technology, Beverly, MA). After incubation, the membranes were rinsed with TBST (10 mM Tris, pH 7.5, 150 mM NaCl, and 0.1% Tween 20). They were then left to incubate for 1 h at room temperature with a secondary antibody conjugated with horseradish peroxidase (Cell Signaling Technology, Beverly, MA). Immunoreactive protein bands were detected using Immobilon Western Chemiluminescent HRP Substrate following TBST washing (Millipore Corporation, USA).

### 2.8 Statistical Analysis

The data are presented as the mean ± standard deviation (SD). One‐way analysis of variance (ANOVA) and Tukey's post hoc test were used to examine statistically significant differences between groups. Statistical significance was determined by utilizing the SPSS statistical software package (version 20.0) and *p* values < 0.05 and < 0.01.

## 3 Results

### 3.1 Effect of Supplement on Cells Proliferation Inhibition

**FIGURE 1:**
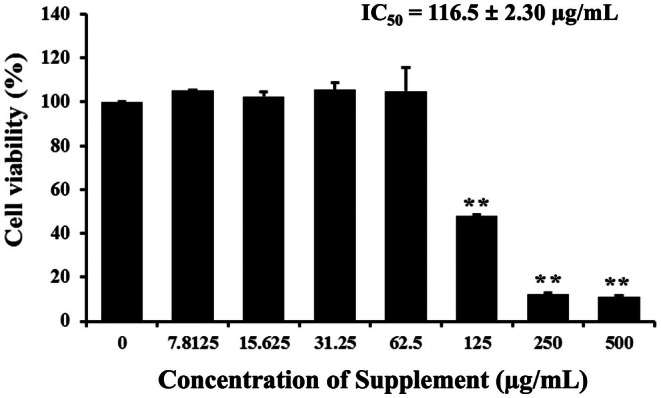
Effects of supplement on cells proliferation inhibition in SK‐BR‐3 cells. SK‐BR‐3 cells were treated with various concentrations of supplement for 24 h and examined by using MTT assay. The percentage of cell viability was showed as mean values ± SD. *p* < 0.01 is considered a significant difference compared to the control.

We first investigated the cell proliferation inhibition of supplement on SK‐BR‐3 cells by MTT assay. Cells were exposed to different concentrations of supplement for 24 h. The results showed that supplement inhibited breast cancer cell proliferation on SK‐BR‐3 cells in a dose‐dependent manner, with an IC_50_ value of 116.5 ± 2.30 μg/mL, as shown in Figure 1. A range of concentrations (50, 75, 100, 125, and 150 μg/mL), encompassing values below and above the IC50 was selected to evaluate the dose‐dependent effects on apoptosis.

### 3.2 Effect of Supplement on Nuclear Morphology Changes

**FIGURE 2:**
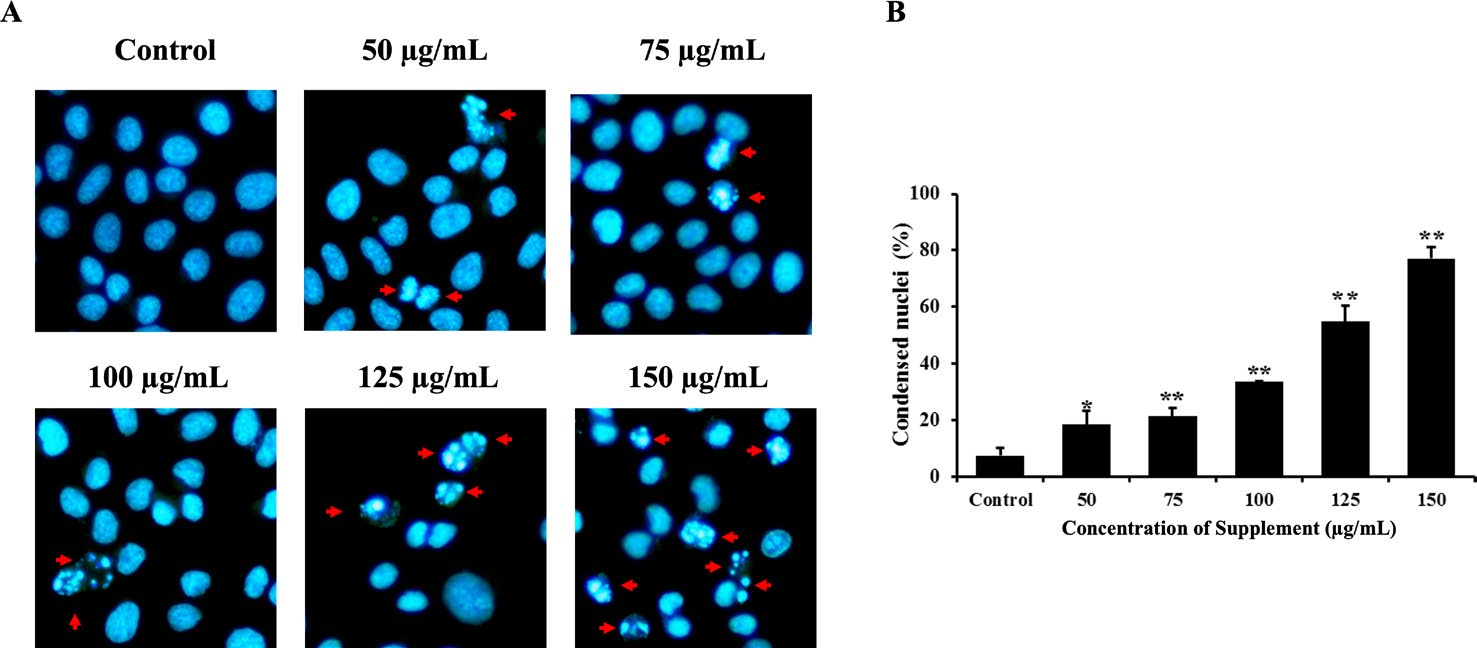
Effects of supplement on nuclear morphological change. (A) The fluorescence images after stained with Hoechst33342 dye (20X). The red arrows present nuclear condensation and fragmentation. (B) The histogram illustrated the proportion of cells exhibiting nuclear condensation relative to the control cells. *p < 0.05 and **p < 0.01 are considered a significant difference compared to the control.

Hoechst 33342 staining was used to detect apoptotic cells with shrunken, condensed, and fragmented nuclei, which were visualized under a fluorescence microscope. As shown in Figure 2, after treatment for 24 h with the supplement, condensed chromatin and nuclear fragmentation increased in a dose‐dependent manner in supplement‐treated cells compared with the control group in SK‐BR‐3 cells. These results indicate that the supplement induces apoptosis in SK‐BR‐3 cells.

### 3.3 Effect of Supplement on Mitochondrial Membrane Potential ( *ΔΨ*m)

**FIGURE 3:**
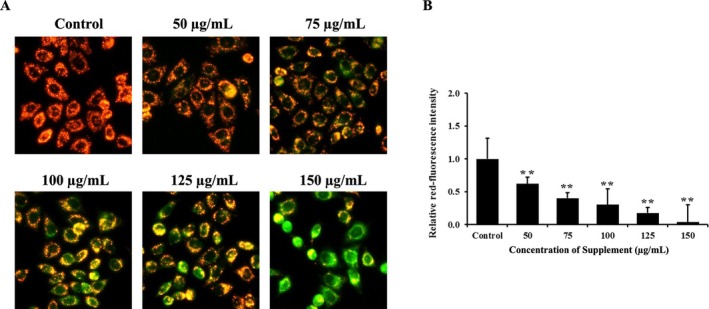
Effects of supplement on mitochondrial membrane potential (*ΔΨ*m) using JC‐1 staining on SK‐BR‐3 cells. (A) The fluorescence images after stained with JC‐1 dye (20X). The green fluorescence signal indicates cells that loss of *ΔΨ*m. (B) The histogram illustrated the red fluorescence intensity relative to the control cells. **p < 0.01 is considered a significant difference compared to the control.

The loss of *ΔΨ*m is one of the major characteristics of cells undergoing apoptosis, which was examined by JC‐1 staining. The results showed that supplement‐treated cells decreased red fluorescence signals, indicating loss of *ΔΨ*m in a dose‐dependent manner, compared with the control group in SK‐BR‐3 cells, as shown in Figure 3. These results indicated that the supplement induces apoptosis in SK‐BR‐3 cells via the mitochondrial pathway.

### 3.4 Effect of Supplement on Cell Cycle Distribution

**FIGURE 4:**
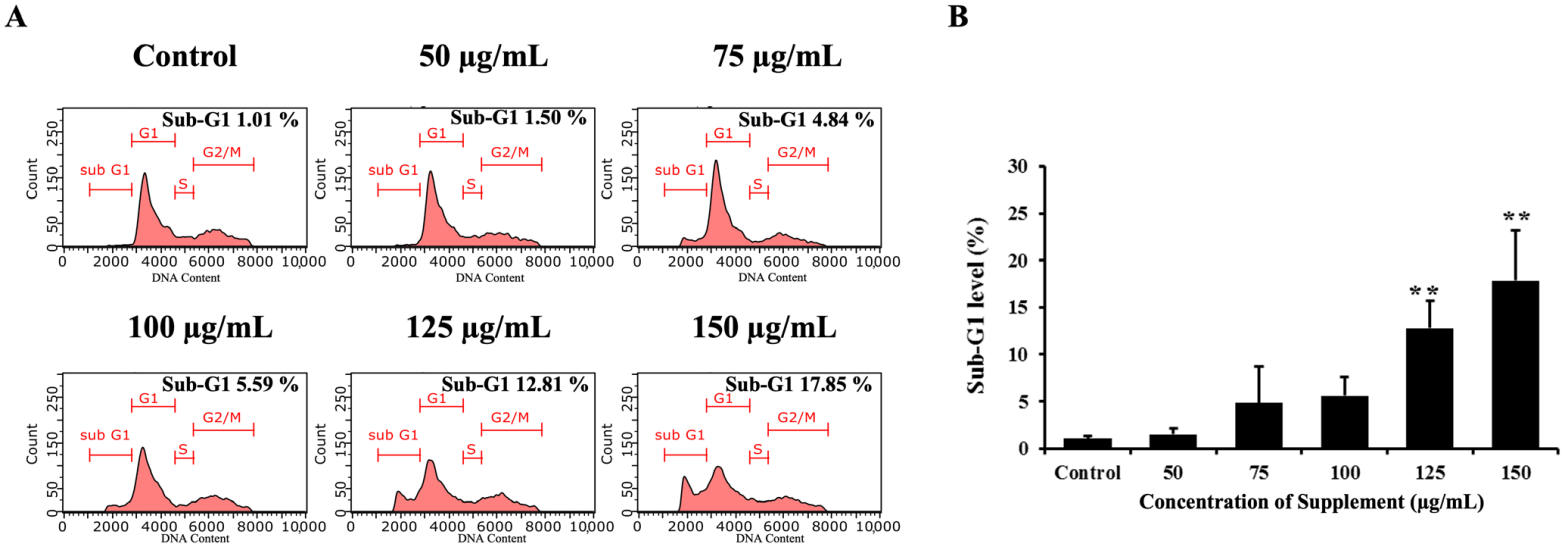
Effect of supplement on cell cycle distribution by flow cytometry analysis on SK‐BR‐3 cells. (A) Representative histograms of cell cycle distribution depicting apoptosis in SK‐BR‐3 cells. The sub‐G1 population indicates presence of apoptotic cells. (B) The histogram illustrated the percentage of sub‐G1 population compared to the control cells. *p < 0.01 is considered a significant difference compared to the control.

One of the markers of apoptosis is DNA fragmentation, which was confirmed by examining changes in the sub‐G1 population of the cell cycle by flow cytometry. We found that supplement increased the sub‐G1 population, indicating genomic DNA fragmentation. SK‐BR‐3 cells treated with supplement at a high concentration (150 μg/mL) showed a maximum sub‐G1 population of 17.85%, as shown in Figure 4. These results revealed that supplement suppresses cell proliferation and triggers apoptosis by promoting the accumulation of sub‐G1 cells.

### 3.5 Effect of Supplement on Apoptosis Induction

**FIGURE 5:**
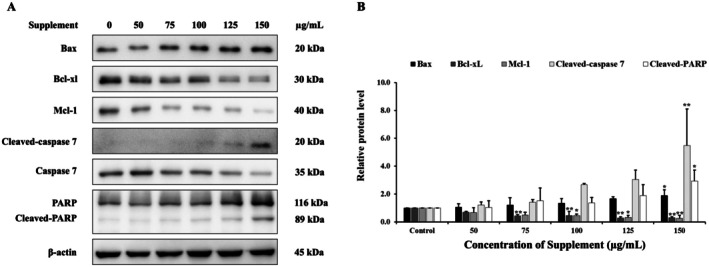
Effects of supplement on apoptosis‐related proteins level in SK‐BR‐3 cells. (A) Protein expression was analyzed using western blot. (B) The relative band intensity of apoptosis‐related proteins was compared to the control cells. β‐Actin served as the internal control. *p < 0.05 and **p < 0.01 are considered a significant difference compared to the control.

The mechanisms of apoptosis induction by supplement were examined by western blot analysis. We investigated whether supplement affects the expression of apoptosis‐related proteins. As illustrated in Figure 5, supplement increased cleaved‐caspase 7 and cleaved‐PARP. Additionally, supplement also increased pro‐apoptotic proteins such as Bax, and decreased anti‐apoptotic protein levels of Bcl‐xL and Mcl‐1 in SK‐BR‐3 cells. Therefore, these findings suggest that supplement induced apoptosis in SK‐BR‐3 cells via the intrinsic apoptotic pathway.

### 3.6 Effect of Supplement on PI3K/Akt Signaling Pathway

**FIGURE 6:**
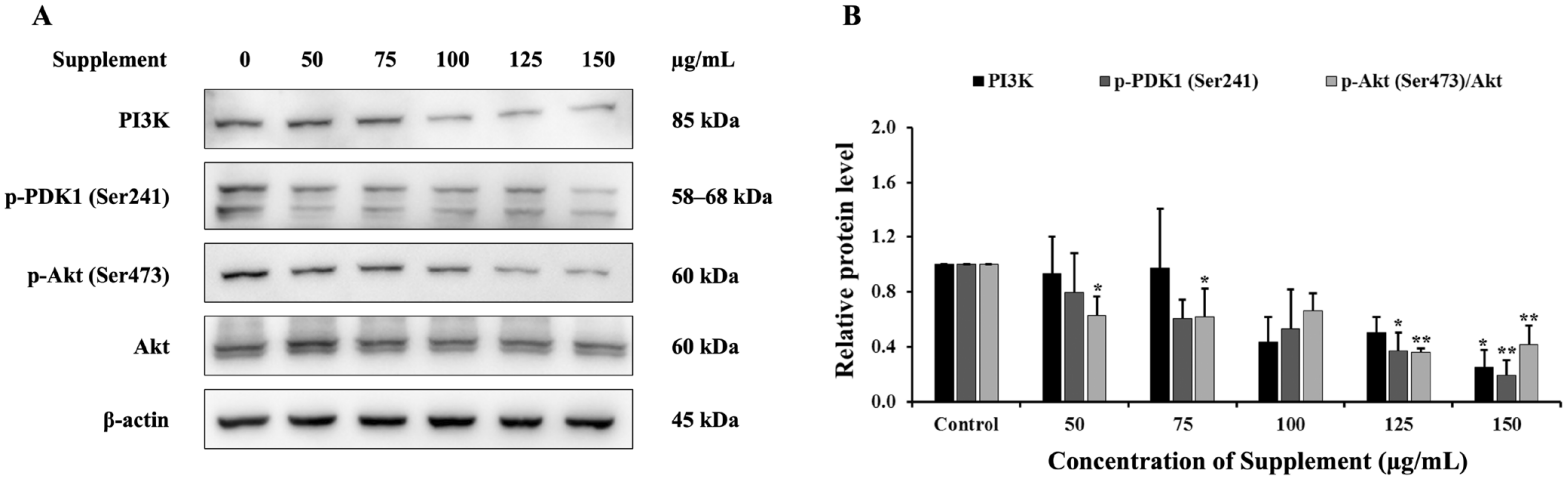
Effects of supplement on PI3K/Akt signaling pathway in SK‐BR‐3 cells. (A) Protein expression was analyzed using western blot. (B) The relative band intensity of PI3K/Akt pathway proteins was compared to the control cells. β‐Actin served as the internal control. *p < 0.05 and **p < 0.01 are considered a significant difference compared to the control.

The results showed that the supplement decreased protein levels of PI3K, p‐PDK1 (Ser241) and p‐Akt (Ser473) compared with the control group in SK‐BR‐3 cells, as shown in Figure 6. These results indicate that the supplement suppressed the cell survival pathway, the PI3K/Akt signaling pathway, in SK‐BR‐3 cells.

### 3.7 Effect of Supplement on MAPK Signaling Pathway

**FIGURE 7:**
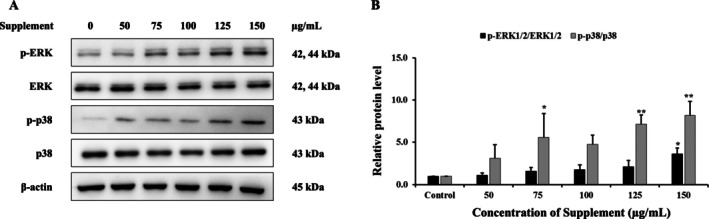
Effects of supplement on MAPK signaling pathway in SK‐BR‐3 cells. (A) Protein expression was analyzed using western blot. (B) The relative band intensity of p‐ERK1/2, ERK1/2, p‐p38 and p38 proteins was compared to the control cells. β‐Actin served as the internal control. *p < 0.05 and **p < 0.01 are considered a significant difference compared to the control.

The results showed that supplement increased the protein expression levels of phosphorylated ERK1/2 and p38 compared with the control group in SK‐BR‐3 cells, as shown in Figure 7. These results indicate that supplement may induce apoptosis in SK‐BR‐3 cells, associated with the activation of the ERK1/2 and p38 MAPK signaling pathway.

## 4 Discussion

The aim of this study was to examine the potential of edible plants as anti‐breast cancer agents in breast cancer cells with HER2 overexpression. Most edible plants are typically grown for their nutritional benefits and are known as vegetables. However, numerous studies have reported the potential impact of bioactive compounds or phytochemicals derived from edible plants in the prevention and treatment of diseases (*Hossain* et al. 2025). The combination of five types of edible plants consists of: (1) *Sesamum indicum* L. or black sesame, which contains lignans such as sesamin, sesamol, sesaminol, and sesamolin (*Wu* et al. 2019), (2) *Psidium guajava* L. or guava, which contains phytochemicals such as phenolic compounds like flavonoids, lycopene, and terpenes (*Naseer* et al. 2018), (3) *Centella asiatica* (L.) Urb. or asiatic pennywort, which contains major active components such as asiatic acid, madecassic acid, asiaticoside, and madecassoside (*He* et al. 2023), (4) *Glycine max* (L.) Merr. or soybean, which contains constituents such as isoflavones, lunasin, and saponin (*Idowu* et al. 2019) and (5) *Garcinia mangostana* L. or mangosteen, which contains more compounds; α‐mangostin and γ‐mangostin are the main bioactive xanthone compounds found in mangosteen pericarp (*Masullo* et al. 2022). These bioactive compounds have been evaluated and suggested for their numerous biological functions, such as antioxidant, anti‐inflammatory, anti‐microbial, and anti‐cancer properties. For example, α‐mangostin, a compound extracted from mangosteen, has been shown to reduce the viability of human breast cancer MCF‐7 cells (*Wathoni* et al. 2021). Supporting our results, we found that the supplement reduced cell viability in the human breast cancer SK‐BR‐3 cell line.

Next, we investigated the effect of the supplement on apoptosis induction in SK‐BR‐3 cells. The findings revealed that the supplement triggered nuclear condensation, the formation of apoptotic bodies, and a decline in mitochondrial membrane potential, all of which are characteristic indicators of apoptosis. Apoptosis is a form of programmed cell death consisting of two main pathways, including extrinsic and intrinsic pathways. The loss of mitochondrial membrane potential, leading to a loss of membrane permeability, is characteristic of the intrinsic or mitochondrial pathway. These events involve Bcl‐2 family proteins resulting in the release of apoptotic effectors such as cytochrome C that activate initiator caspases 9. Both pathways activate executioner caspases 3/7, which cleave target substrates such as PARP, leading to morphological changes associated with apoptosis (*Mashimo* et al. 2021; *Mustafa* et al. 2024). Therefore, we further examined apoptosis‐related proteins to investigate the molecular mechanism. The results showed that the supplement induces apoptosis by regulating both anti‐apoptotic and pro‐apoptotic proteins, as well as activating caspase 7 and inactivating PARP in SK‐BR‐3 cells. These results suggested that the supplement induces apoptosis via the intrinsic or mitochondrial pathway. Previous studies showed that red guava extracts induce apoptosis through caspase 3 activation and PARP cleavage in TNBC cells (MDA‐MB‐231 and MDA‐MB‐468 cells) (*Liu* et al. 2020). Furthermore, α‐mangostin isolated from mangosteen demonstrated cytotoxic effects on SK‐BR‐3 breast cancer cells and exhibited the presence of apoptotic bodies (*Moongkarndi* et al. 2014).

There are several signaling pathways that control apoptosis, including the PI3K/Akt and MAPK signaling pathways. The PI3K/Akt pathway plays a crucial role in regulating various cellular processes such as cell survival, proliferation, and metabolism. Dysregulation of this pathway is often associated with cancer development and resistance to apoptosis in a variety of cancer cell types (*Jiang* et al. 2020; *Li* et al. 2023). Several plant extracts and their phytochemicals have been found to modulate the PI3K/Akt signaling pathway, either by inhibiting the activation of PI3K, blocking Akt phosphorylation, or affecting downstream targets that contribute to apoptosis (*Issinger and Guerra* 2021). For example, asiatic acid, the main ingredient in *Centella asiatica* (L.) Urb., has been demonstrated to stimulate the production of the tumor suppressor protein Pdcd4 by blocking the PI3K/Akt/mTOR/p70S6K signaling pathway. This modulation results in the induction of apoptosis in colon cancer cells (*Hao* et al. 2018).

MAPK pathway is one of the key pathways targeted for cancer prevention and treatment. Activation of MAPK pathway consists of a three‐tiered kinase core: MAP3K activates MAP2K, which activates MAPKs including ERK1/2, JNK1/2, and p38, resulting in modulating cell survival, differentiation, and apoptosis (*Guo* et al. 2020). JNK1/2 and p38 pathways are typically pro‐apoptotic, promoting apoptosis through the activation of pro‐apoptotic proteins, whereas ERK1/2 is generally pro‐survival (*Nadel* et al. 2023; *Porras* et al. 2004). A previous study suggested that α‐Mangostin extracted from mangosteen inhibited cell proliferation, caused DNA fragmentation, and induced nuclear condensation. It also decreased the expression of anti‐apoptotic proteins Bcl‐2 and Mcl‐1 while increasing levels of cleaved caspase 3 and caspase 9. Additionally, the phosphorylation of ERα, PI3K, Akt, and ERK1/2 was also decreased by α‐Mangostin, although p‐JNK1/2 and p‐p38 were increased in human breast cancer T47D cells (*Kritsanawong* et al. 2016).

Although p‐ERK1/2 is traditionally associated with cell proliferation and survival, sustained activation can promote apoptosis by influencing mitochondrial integrity. Specifically, p‐ERK1/2 has been shown to interact with members of the Bcl‐2 family, modulating mitochondrial outer membrane permeabilization (MOMP) and facilitating cytochrome c release (*Cagnol and Chambard* 2010; *Cook* et al. 2017; *Sugiura* et al. 2021). Our findings demonstrated that supplement‐treated SK‐BR‐3 cells exhibited a dose‐dependent reduction in red fluorescence following JC‐1staining, indicating a significant loss *ΔΨ*m. This mitochondrial depolarization is a hallmark of early apoptosis and supports the hypothesis that the supplement induces apoptosis through the mitochondrial pathway. This finding aligns with previous studies suggesting that cannabidiol (CBD), a bioactive compound derived from *Cannabis sativa* , induces apoptosis in colorectal cancer (CRC) cells by downregulating anti‐apoptotic proteins. Additionally, CBD has been shown to activate JNK, p38, and ERK pathways, implying that its pro‐apoptotic effects in CRC cells may be mediated through activation of the MAPK signaling pathway (*Kim* et al. 2024). Taken together, the data suggest that p‐ERK1/2 may shift from its classical pro‐survival role toward promoting apoptosis in this context, likely through mitochondrial membrane permeabilization. This functional switch highlights the complexity of ERK signaling and underscores the importance of cellular context, signal duration, and intensity in determining ERK‐mediated outcomes.

Furthermore, both the PI3K/Akt and MAPK pathways, specifically the RAS/RAF/MEK/ERK cascade, are the major downstream pathways of HER2, which are critical for promoting cell survival, proliferation, and resistance to therapy (*Pan* et al. 2024). The study has reported that α‐mangostin exerts anti‐cancer activity in human breast cancer T47D cells by inhibiting HER2 activation and the downstream PI3K/Akt and ERK signaling pathways (*Kritsanawong* et al. 2016). Drugs such as trastuzumab and lapatinib exert their effects primarily through direct inhibition of HER2 activity, leading to downstream suppression of both the PI3K/Akt and MAPK pathways, ultimately resulting in reduced proliferation and enhanced apoptosis (*Pan* et al. 2024). Although phosphorylated HER2 (p‐HER2) was below the detection limit in the current study (data not shown), the supplement appeared to suppress PI3K/Akt pathway components while modulating ERK1/2 phosphorylation. This suggests that the supplement may exert its effects by acting downstream of HER2, thereby reducing cell proliferation and promoting apoptosis. Overall, these findings suggest that the supplement may function as a multi‐targeted therapeutic agent, disrupting survival signaling through PI3K/Akt inhibition and simultaneously promoting apoptotic pathways via MAPK modulation.

However, this study investigated the effects of the supplement in vitro using a cell culture model. Therefore, further studies using animal models or in vivo are necessary to evaluate the supplement's efficacy in a physiological context and confirm its therapeutic potential. Additionally, future research should explore the combination of the supplement with conventional chemotherapy or HER2‐targeted therapies to assess possible synergistic effects. Such investigations may support the use of natural dietary supplements as adjunctive treatments in HER2‐positive breast cancer, potentially enhancing therapeutic outcomes.

## 5 Conclusion

In conclusion, our results suggest that the supplement inhibits cell proliferation and induces apoptosis in SK‐BR‐3 cells through activation of the intrinsic (mitochondrial) apoptotic pathway. This effect is mediated by the suppression of the PI3K/Akt signaling pathway and the activation of the ERK1/2 and p38 MAPK signaling pathways. These results indicate the potential of Mylife/Mylife100, a Thai innovation, as a dietary supplement that could be further explored as an adjuvant therapy for HER2+ breast cancer. Furthermore, further research is needed to determine the clinical and in vivo efficacy of dietary natural products and their bioactive ingredients in patients with breast cancer.

## Author Contributions

**Pornnapa Sitthisuk:** data curation (equal), formal analysis (equal), investigation (lead), methodology (equal), writing – original draft (lead). **Nichakarn Kwankaew:** investigation (supporting), validation (equal), writing – review and editing (supporting). **Sukanda Innajak:** conceptualization (supporting), investigation (equal), methodology (equal). **Kanokwan Nilchad:** formal analysis (equal), validation (equal), writing – review and editing (supporting). **Watcharaporn Poorahong:** conceptualization (equal), methodology (equal). **Patiwat Kongdang:** validation (equal), writing – review and editing (supporting). **Ramida Watanapokasin:** conceptualization (lead), data curation (equal), funding acquisition (lead), project administration (lead), resources (lead), supervision (lead), validation (equal), visualization (lead), writing – review and editing (lead).

## Ethics Statement

The authors have nothing to report.

## Conflicts of Interest

Asian Phytoceuticals Public Company limited provided the Mylife/Mylife100 supplement and has no influence on the study.

## Data Availability

The data that support the findings of this study are available from the corresponding author upon reasonable request.
